# Supplementary material for: Greater improvement in aerobic capacity after a polarized training program including cycling interval training at low cadence (50–70 RPM) than freely chosen cadence (above 80 RPM)
Source: PLoS One. 2024 Nov 13;19(11):e0311833. doi: 10.1371/journal.pone.0311833 (PMC11559993; doi:10.1371/journal.pone.0311833)
Supplement: Attachment — Submitted filename: PONE-D-24-20867.docx [file pone.0311833.s001.docx]

Dear Authors,

I would like to express my gratitude for the opportunity to review this manuscript and congratulate for their efforts in completing this scientific work.

Monitoring training in women is a demanding process due to the specificity of hormonal changes, affecting the progress of physical performance to a greater or lesser extent. The authors carried out the training intervention reliably and in a very thoughtful approach by constructing an 8-week training with SIT, HIIT and low-intensity endurance training (LIT) in well-trained female cyclists. In order to verify the impact of this training performed at high (above 80RPM) and low cadence (50-70RPM), they not only performed a incremental test, but also conducted a verification of this test. The results of the study are clearly presented and the statistics of the study are described in detail.

However, I have a few concerns and suggestions for Authors:

In lines 50-53 referring to the study by Hebisz et all. detail the intensity of the efforts in the respective SIT, HIIT, LIT protocols and give the duration of the LIT effort.

Did you measure the participants' body mass after the training process? In Your results give the VO_2_max value in the unit ml/kg/min. In the POLFC group, statistical significance was observed in VO_2_max (l∙min^-1^)with non-significance relative to body mass (ml∙min^-1^∙kg^-1^), suggesting that changes in body mass may have occurred and that body mass components should be verified in future researches.

In line 278 correct the unit VO_2_max (correctly ml∙min^-1^∙kg^-1^ and l∙min^-1^).

The indicated errors do not affect the essence of this work, which can provide very important guidance in the training process in well-trained female cyclists.

I look forward to hearing from You.

Best regards,

Reviewer.
