# Supplementary material for: Greater improvement in aerobic capacity after a polarized training program including cycling interval training at low cadence (50–70 RPM) than freely chosen cadence (above 80 RPM)
Source: PLoS One. 2024 Nov 13;19(11):e0311833. doi: 10.1371/journal.pone.0311833 (PMC11559993; doi:10.1371/journal.pone.0311833)
Supplement: Attachment — Submitted filename: Response to Reviewer 2.docx [file pone.0311833.s002.docx]

**Reviewer 2**

 Greater improvement in aerobic capacity after a polarized training program including cycling interval training at low cadence (50-70 RPM) than freely chosen cadence (above 80 RPM)

General Comments:
The manuscript presents an investigation into the effects of two polarized training programs (POL) on aerobic capacity in well-trained female cyclists. The study is well-structured and addresses an important aspect of sports science, particularly in optimizing training methodologies for athletes. However, there are several major and minor weaknesses that need to be addressed.

- Thank you very much for your review and the valuable comments.

Major Weaknesses:
1.Sample Size and Generalizability:
•The sample size (n=12 for each group) is relatively small, which may limit the generalizability of the findings. Larger sample sizes would provide more robust results and enhance the external validity of the study.

- At the end of the Discussion section, we have added a paragraph on the methodological limitations of the presented study. The problem of too small group sizes was described there. Nevertheless, we ask the Reviewer to appreciate the fact that the study on polarized training was conducted in a group of 24 female cyclists. We were not able to gather a larger group of women training in cycling. In recent years, women's sports have been reaching an increasingly higher level, which is why we wanted to conduct the study among women.

2.Lack of Detailed Methodological Description:
•The methodology section lacks detailed descriptions of the training protocols, including the duration and intensity of each training session. This information is crucial for reproducibility and for other researchers to apply similar protocols in their studies.

- As suggested, we have improved the description of training protocols by adding more details. We hope that this change will be sufficient.

3.Statistical Analysis:
•While the manuscript reports the use of analysis of variance (ANOVA), it does not provide sufficient detail on the assumptions of the ANOVA being met (e.g., normality, homogeneity of variances). Additionally, the post-hoc tests used are not mentioned, which is essential to understand the pairwise comparisons.

- The Statistical analysis section included information about the Shapiro-Wilk test. It was also written that in the case of RER-rec, the requirement of similarity to the normal distribution was not met. However, we did not perform an analysis of variance homogeneity. This has been corrected in the current version of the manuscript. Levene's test showed that VT2 did not meet the requirement of variance homogeneity. Therefore, we used the Friedman nonparametric test to analyze RER-rec and VT2.

In the case of ANOVA analysis, the Scheffe test was used as a post-hoc test.

In Table 2, the statistical probability level p for RER-rec was incorrectly marked. This error has been corrected.

Minor Weaknesses:
1.Writing Quality:
•There are several grammatical errors and awkward phrasings throughout the manuscript that need to be addressed to improve readability and clarity.

- The text of the manuscript was checked and some sentences were corrected by a translator with whom our University cooperates.

2.Literature Review:
•The introduction could benefit from a more comprehensive review of the existing literature on polarized training and its effects on different populations, not just cyclists. This would provide a broader context for the study.

- In the Introduction section, a paragraph was added regarding the impact of a polarized training program on the physical capacity and performance of athletes in other sports disciplines.

3.Ethical Considerations:
•While the ethical approval is mentioned, there is no discussion on how the participants' well-being was monitored during the training sessions. This is important to ensure the safety and ethical treatment of participants.

- In the Materials and Methods section, we added information that each participant had to have consent to participate in training from a sports medicine physician. In addition, each participant could withdraw from the experiment at any time without giving a reason. In the event of observing any ailments (e.g. hypertension, tachycardia, dizziness, shortness of breath) – participants were to withdraw from further participation in the experiment.

- In addition, to prevent overreaching and ensure the well-being of the participants, recovery microcycles were used in which the cyclists reduced the volume of the main part of the training session by 50%, similar to the assumptions of undulating periodization of training (Holmes et al. 2018). This is shown in the new figure (Figure 1). We did not include this information in the first version of the manuscript because we thought it would add confusion to the description of the training intervention.

Specific Comments:
1.Title and Abstract:
•The title is clear and informative. However, the abstract should mention the specific duration and intensity of the training programs to provide a complete overview (Lines 16-34).

- As suggested, additional information has been added.

2.Introduction:
•The introduction provides a good rationale for the study but lacks a detailed review of previous studies on polarized training in different populations. Including this would strengthen the background (Lines 39-40).

- In the Introduction section, a paragraph was added regarding the impact of a polarized training program on the physical capacity and performance in different populations

3.Methods:
•The description of the training protocols (Lines 18-22) is too brief. Include details on the frequency, duration, and intensity of the SIT, HIIT, and LIT sessions.

- As suggested, information about the frequency, intensity and duration of SIT, HIIT and LIT sessions has been added.

•Specify the criteria used to select the well-trained female cyclists (Lines 17-18).

- As suggested, the criteria were specified: "The participants of the experiment were considered as well-trained based on their baseline VO2max and training experience, this classification was presented by Decroix et al. (2016)."

They are mentioned in the Abstract and in the Materials and Methods section.

4.Results:
•The statistical analysis section should mention the specific post-hoc tests used for pairwise comparisons (Lines 26-31).

- This has been corrected by mentioning specific tests: "Post-hoc Scheffe analysis"

•Provide a table summarizing the main results for ease of interpretation.

- We are very sorry, but we do not understand this comment. The main results are in table 2 and if we create a new table we will duplicate these results.

5.Discussion:
•The discussion should address the potential limitations of the small sample size and how it might affect the generalizability of the findings (Lines 32-34).

- As suggested, we addressed the limitation related to the small sample size in the final part of the discussion.

6.Conclusion:
•The conclusion is concise but should reiterate the practical implications of the findings for training programs in cyclists (Lines 32-34).

- As suggested, we have added a practical implication in the Conclusion section of the manuscript body: "The findings of the presented study indicate practical application for athletes and coaches in cycling. In the training process, they should take into account not only the intensity and duration but also the cadence used during various interval training sessions, as this may have an additional impact on improving aerobic capacity."

And a shorter version in the Abstract: "This finding is a practical application for athletes and coaches in cycling, to consider not only the intensity and duration but also the cadence used during various interval training sessions."
